# Mechanical Properties and In Vitro Digestibility of Fermented Lentil and Quinoa Flour Food Prototypes for Older Adults

**DOI:** 10.3390/nu16234006

**Published:** 2024-11-23

**Authors:** Elena Gomez-Gomez, Joaquim Calvo-Lerma, Mónica González, Ana Heredia, Amparo Tárrega, Ana Andrés

**Affiliations:** 1 FoodUPV, Universitat Politècnica de València, Camino de Vera s/n, 46022 Valencia, Spain; anhegu@tal.upv.es (A.H.); aandres@tal.upv.es (A.A.); 2 Research Group in Innovative Technologies for Sustainable Food (ALISOST), Faculty of Pharmacy and Food Sciences, Universitat de València, Avda, Vicent Andrés Estellés, 46100 Burjassot, Spain; joaquim.calvo@uv.es; 3 Instituto de Agroquímica y Tecnología de los Alimentos (IATA-CSIC), Avda, Agustín Escardino Benlloch 7, 46980 Paterna, Spain; m.gonzalez@iata.csic.es (M.G.); a.tarrega@iata-csic.es (A.T.)

**Keywords:** plant based, solid-state fermentation, *Pleurotus ostreatus*, older adult, legumes, pseudo-cereals

## Abstract

**Background/Objectives**: The increase in the older adult population worldwide and the need to switch to vegetal-origin protein consumption for environmental sustainability point to legumes and pseudocereals as alternative ingredients in new food formulations. This study aimed to assess the impact of food structure and fungal fermentation on the digestibility of new food prototypes made with quinoa and/or lentil flours addressed to older adults. **Methods**: Four gels and six breads were elaborated and subjected to mechanical analysis and simulated gastrointestinal in vitro digestion. Then, proteolysis, lipolysis, and amylolysis were analysed. **Results**: Gels made with fermented quinoa or lentil flours exhibited less hardness and required less force, suggesting better adequacy for mastication. In terms of digestibility, using fermented flours led to increased proteolysis and reduced starch hydrolysis. **Conclusions**: Our results support future studies in the field aimed at supplying older adults with adapted foods to satisfy their nutritional needs to prevent sarcopenia and other health issues.

## 1. Introduction

The World Health Organization (WHO) estimates that the proportion of older adults in the global population will double, reaching 22% by 2050 [1]. This demographic shift presents a challenge to the scientific community, which must focus on promoting quality ageing. Central to this is addressing nutritional issues related to diet, with an emphasis on adequate protein intake. Additionally, protein transition has been identified as a key strategy for mitigating climate change by reducing animal protein consumption in favour of plant-based proteins [2].

However, the transition to plant-based proteins poses challenges due to their lower nutritional quality and digestibility compared to animal-derived proteins. This is particularly concerning for older adults, who often experience maldigestion and malabsorption. Ageing is associated with physiological changes in the gastrointestinal tract, which are compounded by medication use, which predisposes older individuals to disruptions in the digestive process [3,4]. These challenges highlight the importance of creating specific food products tailored to the nutritional needs of this demographic. Key concerns include protein and essential nutrient absorption deficiencies, leading to muscle mass loss (sarcopenia) and increased insulin resistance [5,6,7].

A marked increase in animal protein consumption has been observed over the past 50 years, accounting for more than half (58%) of the per capita daily protein intake [8]. At this rate, meat production would need to increase by 200 million tonnes to meet projected demand by 2050, which is environmentally unsustainable [9].

Therefore, alternative and sustainable protein sources are needed to ensure adequate and high-quality protein intake. Among current alternatives to animal protein, legumes and pseudo-cereals, such as lentils and quinoa, stand out as valuable sources of plant-based protein, not only due to their environmental sustainability and low cost but also because of their high fibre, mineral, and vitamin content, as well as their antioxidant properties or content of beneficial phytochemicals among other benefits [10,11]. Despite these benefits, plant-based materials typically contain antinutritional compounds (e.g., phytates, protease inhibitors, lectins, tannins) that may limit their nutritional value by hindering protein digestibility and reducing the bioavailability of minerals [12,13]. In this context, solid-state fermentation with the fungus *Pleurotus ostreatus* has been investigated as an effective method for reducing phytates, increasing protein content and digestibility, and enhancing resistant starch [11,13,14,15]. As a result, solid-state fermentation, followed by drying and milling, could be used to produce fermented plant protein-rich flours and also improve their antioxidant capacity and phenolic profile. These fermented flours could serve as ingredients in new food products designed to meet the nutritional needs of older adults while promoting environmental sustainability. When designing such food products, the structure of the food matrix is an important consideration. For older adults, foods should be easy to chew and swallow, provide sensory appeal [16,17], and be digestible [10,18].

Thus, this study aimed to evaluate the impact of fungal solid-state fermented lentil and quinoa flours in high and low-water content prototypes (gel and bread, respectively) on the textural characteristics and in vitro macronutrient digestibility under gastrointestinal conditions representative of the older adult population.

## 2. Materials and Methods

### 2.1. Materials and Reagents

Lentil grains (*Lens culinaris*, Castellana variety) and quinoa seeds (*Chenopodium quinoa* Wild, white variety) were purchased at a local supermarket in València (Spain). The other ingredients were wheat flour (Harinera del Mar Siglo XXI S.L., Castellón, Spain), instant yeast (Cortés Bartolomé S.L., Valladolid, Spain), sugar (Zukán S.L.U., Murcia, Spain), salt (A. Martínez, Cheste, Spain), extra virgin olive oil (EVOO) (Fontoliva, Jaén, Spain) apple cider vinegar (Hacendado, València, Spain), bicarbonate (A. Martínez, Cheste, Spain), and natural mineral water (Aguas de Cortes S.A., Castellón, Spain).

Fermented flours were obtained by solid-state fermentation with the edible fungus *Pleurotus ostreatus*, followed by air-drying at 70 °C, with an air humidity percentage of 8.7 ± 1.2, and milling using a food processor (Vorwerk, Thermomix^®^ TM6-1, Wuppertal, Germany), applying 10,000 rpm at 15 s intervals for 1 min. Unfermented flours of lentils and white quinoa (as controls) were obtained by milling commercial grains under the same conditions. The procedure was based on the protocol by Sánchez-García et al., 2023 [15,19], where the proximal composition of lentil and white quinoa flours, both unfermented and fermented, can also be found.

### 2.2. Prototype Development

A total of 10 new food prototypes were formulated. On the one hand, six gel prototypes were prepared using flours from different raw materials (quinoa, lentil, and a quinoa-lentil combination), both fermented and unfermented. A kitchen robot (Mambo 8090, Cecotec, Spain) multifunction food processor was used to mix all the ingredients (flour, water, salt, oil, and either bicarbonate or vinegar) for 15 min at 90 °C and a speed of 4. The mixture was then allowed to cool while being stirred at a speed of 4 for an additional 6 min. Once cooled, the mixture was poured into moulds and left to gel for 24 h at 4 °C.

On the other hand, four bread-like prototypes were prepared with wheat flour and enriched with either lentil or a quinoa-lentil flour combination, using both fermented and unfermented flours. Bread enriched solely with quinoa was not included as it was not possible to produce a suitable loaf of bread at the proposed level of wheat replacement (50%). First, the ingredients were mixed with a kitchen blender (Mambo 9090, Cecotec Innovaciones S.L., Valencia, Spain) for 1 min at 1100 rpm, adding the ingredients in the following order: flour, salt, sugar, yeast, water, and oil, ensuring that the water was poured over the yeast to activate it. The water was preheated to a constant temperature of 40 °C using another blender (Thermomix, Vorwerk Spain, M.S.L., Madrid, Spain) before being mixed with the other ingredients. After mixing, the batter was piped into plastic bags, with 80 g of batter placed in each aluminium cup. These were kept at 30 °C for a 1 h fermentation, then baked in a preheated oven (Smeg Model ALFA144GH1, Smeg S.p.A., Guastalla, Italy) at 160 °C for 30 min under humid conditions. The breads were left to cool at room temperature for 1 h before being stored in resealable plastic bags for 24 h prior to analysis the following day.

The formulations (in the percentage of ingredients) of the six gel-like and four bread-like prototypes are presented in Table 1, while their proximate composition, including moisture, protein, fat, fibre, and NaCl (g/100 g), is shown in Table 2. This composition was theoretically calculated based on the nutritional values of the fermented flours previously characterised [15,19].

### 2.3. Mechanical Characterisation of the Prototypes

Textural measurements of gel and bread prototypes were conducted using a texture analyser (TA/XTPlus, Stable Micro Systems Ltd., Surrey, UK).

#### 2.3.1. Gels

Texture profile analysis (TPA) was performed on gel cylinders (height = 22 mm, diameter = 17 mm) using an aluminium probe with a diameter of 75 mm (SMS P/75). Force was registered while the gel cylinder was compressed twice, up to 10% of its original height and at a constant speed of 1 mm/s. From the force–time curves, mechanical parameters were obtained. Hardness was obtained as the maximum force achieved during the first compression cycle. Elasticity corresponded to the % recovery of the product to its original height. Cohesiveness was obtained as the relative resistance to deformation between the two cycles of compression (Area 2/Area 1), excluding the areas under the decompression portion. Measurements were conducted twice, and eight cylinders of two batches were analysed.

Resistance to Penetration: The penetration test was conducted using a 10 mm diameter cylindrical probe. The force was recorded when the sample gel contained in the cylindrical container (height = 2 mm, diameter = 36 mm) was penetrated to 10 mm at 1 mm/s. The parameters obtained were the maximum penetration force and the distance to reach the maximum penetration force. Measurements were conducted twice, and two cylinders per batch were measured.

Cutting Test: Gel cylinders (height = 62 mm, diameter = 36 mm) were cut transversally at a constant speed of 1 mm/s using a butter/wire cutter (A/BC). Two parameters were obtained from the force–time curve: the cutting force (maximum value of force registered) and adhesiveness (a negative area corresponding to the probe’s return to the initial position). Measurements were conducted twice, and two cylinders per batch were measured.

#### 2.3.2. Breads

Texture profile analysis (TPA). Cubes of crumb (20 mm × 20 mm) were obtained from each bread. A double compression was applied to the crumb cube using a 75 mm diameter aluminium platen (P/75) at 1 mm s^−1^ until 40% deformation and with an interval of 5 s between compressions. From the force–time curve registered during the compression, the parameters hardness (first compression’s highest peak force), elasticity (% recovery of the product to its original height), cohesiveness (division between the second and first compression curves’ areas), and chewiness (hardness × cohesiveness × elasticity) were gathered. Two batches of pieces of bread were prepared per formulation, and four cubes were measured per batch.

A penetration test (PT) was carried out on the top part of the bread to evaluate the crumb and crust together. Force was registered when descending a 2 mm diameter cylinder probe (P/2) at 1 mm s^−1^ until 5 mm penetration. Five tests were conducted at five different points of the bread surface. The parameters obtained were the maximum penetration force and the distance to reach the maximum penetration force. Two batches of bread were prepared per formulation, and four pieces of bread were measured per batch.

### 2.4. Gastrointestinal Static In Vitro Digestion

The prototypes were subjected to in vitro simulated gastrointestinal digestion according to the model proposed for older adults by Ménard et al., 2023 [20]. Stock solutions and simulated digestive fluids were prepared in advance following the INFOGEST standardised protocol (Brodkorb et al., 2019) [21], with modifications applied to pH, duration, and pepsin concentration during the gastric stage, as well as pancreatin and bile salt concentrations during the duodenal stage, to better replicate conditions in older adults, as previously described [22].

Oral stage: Simulated salivary fluid (SSF; pH 7) containing alpha-amylase (112.5 U/mL) was mixed with 5 g of each gel and bread prototype in a 1:1 (*v*/*w*) ratio. The mixture was homogenised for 1 min at 8500 rpm using an Ultra-Turrax device (Ultra-Turrax T25D, IKA, Staufen, Germany) and incubated in a thermostatic chamber (JP Selecta SA, 3000957, Barcelona, Spain) at 37 °C for 2 min.

Gastric stage: Simulated gastric fluid (SGF; pH 3.7) was added to the bolus in a 1:1 (*v*/*w*) ratio. Porcine pepsin was added to the SGF to achieve a concentration of 1200 U/mL in the gastric mixture, forming the chyme. The pH was adjusted with Hydrochloric Acid (1N) to 3.7 ± 0.1. Samples were then shaken in an Intell-Mixer RM-2 (Elmi Ltd., Riga, Latvia) at 55 rpm for 3 h at 37 °C. Incubation took place in a chamber (JP Selecta SA, Barcelona, Spain).

Intestinal stage: Simulated intestinal fluid (SIF; pH 7) was added to the chyme in a 1:1 (*v*/*w*) ratio. Pancreatin and bile salts were incorporated into the SIF to reach concentrations of 80 U/mL and 7 mM, respectively, in the intestinal mixture. The pH was adjusted to 7.0 ± 0.1 using Sodium Hydroxide (NaOH, 1 N). Samples were subjected to periodic inversion at 55 rpm for 2 h at 37 °C. Following digestion, the samples were centrifuged at 8000× *g* for 10 min, and aliquots of the bioaccessible fraction were collected for various analytical assays. Three independent digestion runs were performed for each prototype, and samples were stored in Eppendorf tubes at −20 °C until further analysis. All reagents were obtained from Sigma-Aldrich Chemical Company (St Louis, MO, USA).

### 2.5. Macronutrients’ Digestibility

#### 2.5.1. Protein Digestibility

The TCA-soluble protein content of the samples was determined at the end of the gastric and intestinal stages, following the methods described by Gallego et al., 2020 [14]. TCA was added to the digested samples (1 mL) to achieve a final concentration of 12% (*w*/*w*). The mixture was vortexed and incubated for 15 min at 10 °C. Afterwards, the samples were centrifuged at 7168× *g* for 10 min (Eppendorf MiniSpin Plus, Sigma-Aldrich, St. Louis, MO, USA).

The portion of the protein that remained soluble in 12% TCA, which consisted of small peptides and amino acid residues, was considered the digested/hydrolysed fraction, representing the extent of proteolysis. The supernatant was diluted in a buffer solution (50 mM EDTA, 8 M urea, pH 10), and protein concentration was determined by measuring the absorbance at 280 nm using a spectrophotometer (Helios Zeta UV/Vis, Thermo Scientific, Waltham, MA, USA). A calibration curve was created using tyrosine as the standard.

#### 2.5.2. Lipid Digestibility

Following gastrointestinal digestion, the digested samples were diluted 100-fold with a solution containing 5.6% Triton X-100 and 6% ethanol in water at the end of the intestinal stage [23]. This step was essential to dissolve free fatty acids and inhibit lipase activity. The released fatty acids were quantified in the diluted samples using a free fatty acid colorimetric assay kit (Roche Diagnostics, Indianapolis, IN, USA) and a spectrophotometer (UV/Vis, Beckman Coulter, Brea, CA, USA) by measuring the absorbance at 570 nm. Palmitic acid was used as a standard to quantify free fatty acids (FFAs). The extent of lipolysis was expressed as the percentage of total fatty acids theoretically released after complete digestion, assuming the maximum release of two fatty acids per triacylglycerol molecule and an average molecular weight for triolein of 885.432 g mol^−1^.

#### 2.5.3. Amylolysis Extent

The release of monosaccharides was measured using the dinitrosalicylic acid (DNS) colorimetric method after the hydrolysis of starch in the sample with invertase and amyloglucosidase. After the intestinal digestion step, 0.250 mL aliquots of digested samples were taken and mixed with 1 mL ethanol, followed by a 30 min incubation. The samples were then centrifuged (100× *g*, 10 min, 20 °C). After centrifugation, 50 µL of the supernatant was added to 250 µL of an enzyme solution (1% amyloglucosidase and 1% invertase) and 750 µL of DNS solution (1% glucose, 1% NaOH 1 M, and 5% DNS) in an Eppendorf tube. To initiate the colorimetric reaction, the tubes were heated at 100 °C for 15 min and then diluted with 4 mL of distilled water. Spectrophotometric measurements were taken at 530 nm (Helios Zeta UV/Vis, Thermo Scientific), and glucose was used to create a calibration curve (0–10 mg/L).

### 2.6. Statistical Analysis

Data were summarised as mean and standard deviation of at least three replicates. Statgraphics Centurion-XV software was used for the statistical analysis. ANOVA was applied to study the statistical significance of unfermented and fermented flours and structural matrix (gel vs. bread) on the studied variables of texture and macronutrients’ digestibility (proteolysis, lipolysis, and amylolysis). The analyses were conducted with at least a significance of 95% (*p*-value < 0.05).

## 3. Results and Discussion

### 3.1. Mechanical Properties of the Prototypes

Focusing first on the gel prototypes, those developed with fermented flour, whether from quinoa or lentil, exhibited improved textural properties, such as reduced hardness, penetration force, or cutting test force, compared to their unfermented counterparts (Table 3). However, the gels containing a blend of quinoa and lentil flours did not show significant changes in most of the cited textural properties. These findings suggest that gels made with fermented quinoa or lentil flours could be easier to masticate due to their lower hardness [24]. In all prototypes, elasticity and adhesiveness did not significantly vary with fermentation but rather with the type of flour used. Gels made with quinoa flour alone (either fermented or unfermented) were more elastic, cohesive, and less adhesive, indicating a more compact internal structure that resists deformation better than those containing lentil flours, which were more plastic and adhesive.

Moving on to the bread prototypes, the results showed that hardness decreased only in the fermented quinoa + lentil bread, while it significantly increased in the formulation containing only lentils (Table 4). Likewise, the rest of the assessed parameters significantly increased with fermentation, except for force (N). Therefore, the impact of flour fermentation on the mechanical properties differed between the lentil and quinoa gels and the breads enriched with lentils and quinoa.

Fermented flours produced softer and less resistant gels, likely because the starch in the fermented flours was already pre-gelatinised, and the proteins were denatured during the heating steps used in the fermentation process. Under these conditions, the gelation of these two flour components is diminished [25].

Consequently, using fermented flour provides added rheological value by adapting to the needs of the elderly, addressing several common issues such as dental problems, swallowing difficulties, digestive issues, and specific nutritional requirements. Softer foods created with fermented flours are easier to chew and swallow, potentially reducing strain on dental structures and lowering the risk of choking. Moreover, fermentation enhances the digestibility of foods, making them more suitable for sensitive digestive systems and meeting the dietary needs of the elderly.

In the case of bread, enrichment with fermented flour resulted in increased cohesiveness, hardness, and resistance, especially in bread made with lentil flour alone. Softer breads were anticipated when using fermented flours, as pre-gelatinised starch has previously been shown to improve texture (softer and easier to chew) in wheat breads. However, in this case, the increase in cohesiveness and hardness can be attributed to protein denaturation, which inhibits the formation of an appropriate starch–protein matrix needed to retain air bubbles, resulting in smaller bubbles and a dense crumb [26]. This could be due to inadequate amylase activity caused by inactivation at high temperatures, which limits bread volume and results in a rigid starch structure [27]. Another reason could be that breads with fermented flours contain higher protein content and increased hardness has previously been associated with heat-induced aggregation in protein-enriched breads [28].

### 3.2. Protein Digestibility

After simulated gastric and intestinal stages of digestion of the fermented and unfermented food prototypes, the digestibility of proteins, lipids, and starch was assessed. The most significant finding was related to proteolysis, as protein was the key macronutrient under investigation because of its relevance in older adults’ diets to prevent sarcopenia.

**Figure 1:**
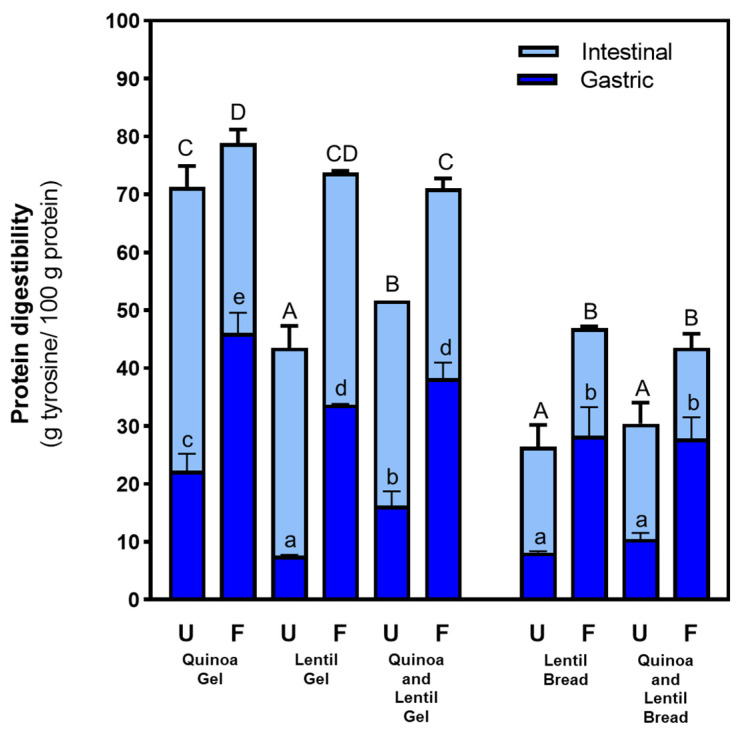
Proteolysis extent (%) of gel-like and bread-like prototypes after gastric and intestinal in vitro digestion under older adult conditions. Different lowercase letters indicate statistically significant differences among prototypes (*p* < 0.05) at the gastric stage. Different capital letters indicate statistically significant differences (*p* < 0.05) among prototypes at the end of digestion. Unfermented (U), Fermented (F).

As shown in Figure 1, protein digestibility was higher in the gels compared to the breads, possibly due to differences in matrix complexity and moisture content. In the gels, the flour components appeared directly dispersed in the water’s continuous phase, likely making them more accessible to pepsin and pancreatin. This is supported by previous studies that highlight nutrient accessibility to enzymes as a key factor in the extent of hydrolysis [29], particularly under sub-optimal intestinal conditions [10]. Furthermore, bread samples with a higher number of ingredients presented a more complex structure, promoting interactions among macronutrients that could hinder pepsin and pancreatin access to proteins during digestion [30,31].

Additionally, prototypes made with fermented flours showed significantly higher protein digestibility compared to those made with unfermented flours. Specifically, the gel and bread prototypes containing fermented lentil flour demonstrated almost a twofold increase in proteolysis by the end of digestion compared to prototypes made with unfermented flours. Gels with fermented flours exhibited lower hardness and cutting force than their unfermented counterparts. However, despite achieving the highest proteolysis values, quinoa formulations showed higher cohesiveness than lentil gels. Therefore, cohesiveness is not a limiting mechanical factor in digestion. Differences in texture among bread prototypes also did not seem to explain variations in protein digestion. Thus, solid-state fermentation emerged as the primary factor affecting protein digestibility, with its impact being more pronounced during the gastric stage and in lentil flour-based prototypes.

These findings highlight the potential of solid-state fermentation to improve the protein digestibility of legume-based food products. Previous studies suggest that fermentation enhances protein digestibility by reducing non-nutritive compounds that inhibit digestive enzymes (e.g., trypsin and chymotrypsin inhibitors) and promoting protein crosslinking (e.g., phenolic and tannin compounds) while also producing microbial proteases that partially hydrolyse proteins within the matrix [10,32].

When comparing quinoa-based to lentil-based formulations, quinoa exhibited higher protein digestibility, which is consistent with previous studies showing that quinoa proteins are highly digestible due to low levels of trypsin inhibitors [33]. Quinoa also contains less insoluble fibre than lentils, which may reduce interactions and favour a less viscous digestion medium [34]. Conversely, lentils have higher levels of anti-nutrients like phytates and tannins that inhibit protein digestibility [10,32,35]. In the digestion stage, fermentation led to increased proteolysis, particularly during the gastric stage, which was equivalent to the intestinal digestion of unfermented samples. This sharp increase in the gastric stage may be attributed to the pre-digested proteins generated by the fungi during fermentation [12] rather than the efficiency of the gastric digestion process itself.

Thus, the consumption of lentil- and quinoa-based foods using fermented flours could help mitigate the reduced protein digestibility observed in elderly digestion models compared to healthy adult models [22,36]. Despite the inherent disadvantage of plant proteins compared to animal proteins in terms of digestibility, fermentation with *Pleurotus ostreatus* seems to offer a biotechnological strategy to improve the digestion and bioavailability of essential nutrients, increasing the health benefits of consuming plant-based proteins.

### 3.3. Lipid Digestibility

**Figure 2:**
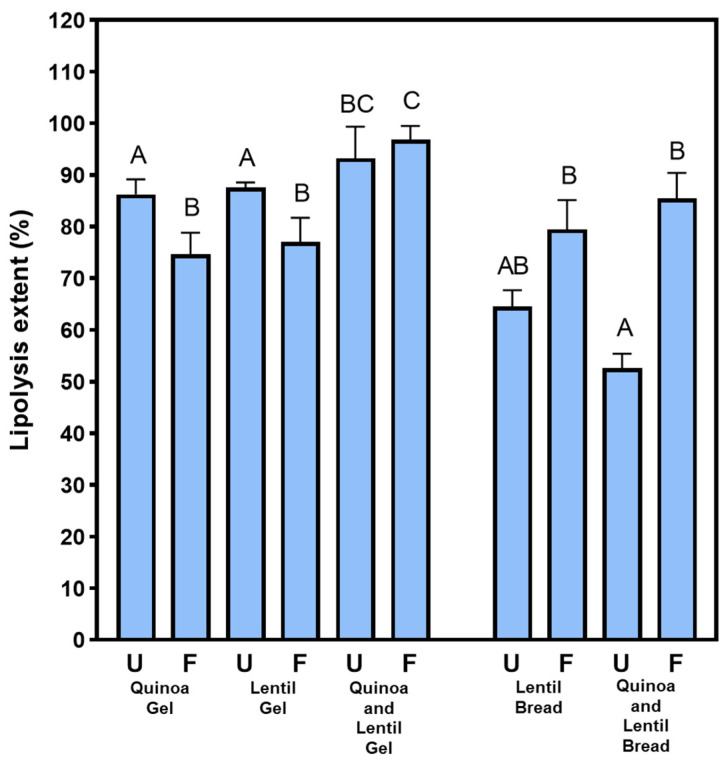
Lipolysis extent (%) of gel-like and bread-like prototypes at the end of in vitro digestion under older adult conditions. Different capital letters indicate statistically significant differences (*p* < 0.05) among prototypes. Unfermented (U), Fermented (F).

Regarding lipolysis extent (Figure 2), the proportion of free fatty acids released ranged from 60 to 90%, being slightly but significantly higher in gels than in bread-like prototypes. Previous studies found reduced lipolysis after in vitro digestion of structured foods [37]. However, lipids in gel structures are easily emulsified by bile salts, resulting in nearly complete lipolysis during the intestinal stage [38]. In addition, protein hydrolysis in gel structures releases peptides and amino acids with excellent emulsifying properties, further promoting lipid digestion and the bioavailability of liposoluble compounds such as free fatty acids, vitamins, antioxidants, or phytosterols [38]. This can be particularly beneficial for elderly individuals if healthy plant fats are used in food formulations.

Another factor influencing fat digestion and absorption is the type of lipids or the triglyceride composition of the fatty acids [39,40]. However, all prototypes used the same lipid component—extra virgin olive oil (EVOO). Therefore, the differences observed may be attributed to the type of flour in the formulation and the effects of solid-state fermentation. Lipids in gels made with fermented flours exhibited slightly but significantly lower lipolysis values than gels with unfermented flours, whereas the opposite trend was observed in bread-like prototypes. Fermented flours display different techno-functional properties compared to unfermented flours [25]. Higher swelling and water-holding capacities, attributed to changes in protein and starch during thermal treatments (autoclaving and drying), may modulate gastrointestinal behaviour and digestibility of the flours.

### 3.4. Starch Digestibility

**Figure 3:**
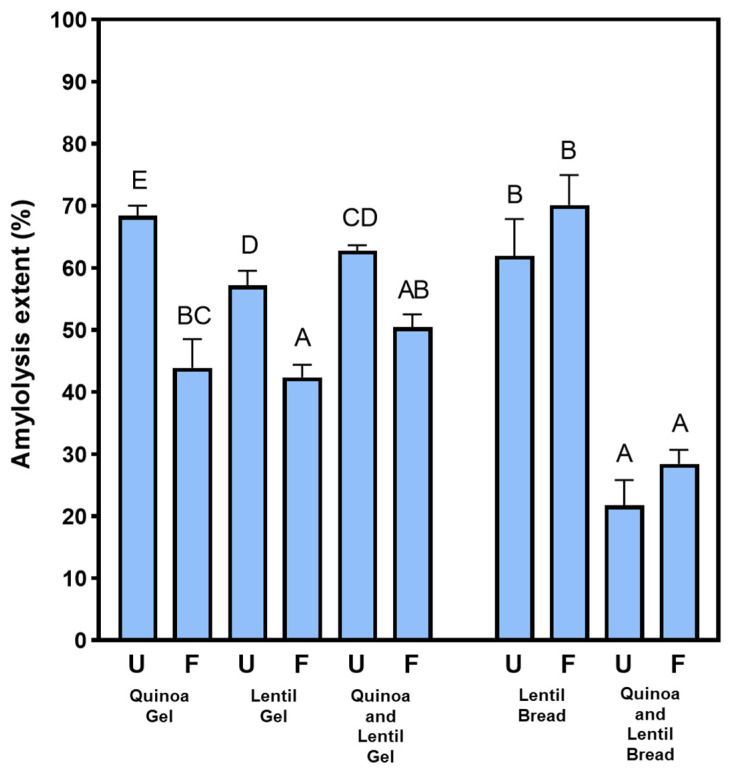
Amylolysis extent (%) of gel-like and bread-like prototypes at the end of in vitro digestion under older adult conditions. Different capital letters indicate statistically significant differences (*p* < 0.05) among prototypes. Unfermented (U), Fermented (F).

Starch hydrolysis (amylolysis) was lower in fermented flours, while no statistically significant differences were observed for bread prototypes (Figure 3). Under optimal fermentation conditions, functional microorganisms can reduce glycaemic responses and improve the dietary fibre complex in food products [41]. Starch digestibility is influenced by various factors, such as the binding of α-amylase to the substrate, gastric emptying, enzyme inhibitors, and the properties of digestive enzymes [42,43]. Processing conditions like heat treatment, cooking, or fermentation also affect starch microstructure by breaking down crystalline structures and promoting retrogradation, resulting in resistant starch that is less digestible [44,45].

Solid-state fermentation may serve as an effective strategy to modulate starch digestibility and reduce the glycaemic index of quinoa and lentil-based products. Studies have shown that Pleurotus species preferentially metabolise starch as a carbon source during solid-state fermentation [12]. Starch metabolisation, coupled with organic acid production, is co-responsible for reduced starch hydrolysis by digestive enzymes. Additionally, pre-gelatinisation and retrogradation of starch during fermentation promote the formation of resistant starch and increase fibre content [25], both of which contribute to decreased digestibility. This hypoglycaemic effect may provide additional value to fermented flour gels, potentially helping to prevent type 2 diabetes, which often develops with age.

Before reaching the conclusions, some limitations should be acknowledged. This study would have benefited from a sensory analysis to assess the acceptance of the prototypes by older adults. However, at this first step of food concept or prototype development, the use of bicarbonate and apple cider vinegar in the formulations prevented us from conducting this type of analysis. Therefore, the formulation of these novel food prototypes will be re-evaluated in the next steps of the scale-up process to optimise sensory characteristics, enabling relevant sensory analyses.

## 4. Conclusions

This study demonstrates that two new food prototypes made with fermented quinoa and lentil flours exhibit improved textural properties, particularly in gel formulations, compared to their unfermented counterparts. This suggests that these novel fermented plant-based ingredients could play a key role in developing foods that are easier to chew and swallow, specifically adapted for older adults. Additionally, both the bread and gel prototypes formulated with fermented quinoa and lentil flours showed enhanced digestibility of proteins compared to those made with unfermented flours. In the case of gels, starch hydrolysis was significantly reduced during in vitro digestion trials, although no such effect was observed in the bread formulations.

In conclusion, these findings support the advancement of research aimed at providing elderly populations with new food products tailored to meet their nutritional requirements.

## Figures and Tables

**Table 1:** Ingredients composition of six gel-like and four bread-like prototypes (expressed in percentage (%)).

Ingredients	UQ-G/FQ-G	UL-G/FL-G	UQL-G/FQL-G	UL-B	FL-B	UQL-B	FQL-B
Water	67.3%	68.8%	70.3%	38.37%	44%	38.37%	38.37%
Quinoa Flour	25%	-	12.5%	-	-	12.92%	11.73%
Lentil Flour	-	25%	12.5%	25.83%	23.47%	12.92%	11.73%
Wheat Flour	-	-	-	25.83%	23.47%	25.83%	23.47%
EVOO	4%	4%	4%	5.9%	5.36%	5.9%	5.9%
Vinegar	3%	-	-	-	-	-	-
Baking Soda	-	1.5%	-	-	-	-	-
Sugar	-	-	-	1.85%	1.67%	1.85%	1.85%
Instant Yeast	-	-	-	1.04%	1.04%	1.04%	1.04%
Salt	0.7%	0.7%	0.7%	1.18%	1.08%	1.18%	1.18%

Unfermented Quinoa Gel (UQ-G), Fermented Quinoa Gel (FQ-G), Unfermented Lentil Gel (UL-G), Fermented Lentil Gel (FL-G), Unfermented Quinoa Lentil Gel (UQL-G), Fermented Quinoa Lentil Gel (FQL-G), Unfermented Lentil Bread (UL-B), Fermented Lentil Bread (FL-B), Unfermented Quinoa Lentil Bread (UQL-B), Fermented Quinoa Lentil Bread (FQL-B).

**Table 2:** Proximate composition (g/100 g of flour) of the six gel-like and four bread-like prototypes made with unfermented and fermented lentils and/or quinoa flours.

Prototypes		Moisture	Protein	Fat	CHO	Fibre	NaCl
Gels (G)	UQ-G	72.3	3.2	5.6	17.7	1.7	0.7
	FQ-G	71.6	4.3	4.6	18.9	3.6	0.7
	UL-G	70.9	6.0	4.3	15.8	4.0	0.7
	FL-G	70.5	7.2	4.5	15.7	3.6	0.7
	UQL-G	72.4	4.6	5.0	16.7	2.8	0.7
	FQL-G	71.8	5.7	4.5	17.3	3.6	0.7
Breads (B)	UL-B	37.9	9.9	7.5	42.4	5.4	1.3
	FL-B	42.4	10.4	7.1	38.9	4.6	1.2
	UQL-B	37.9	8.3	8.3	36.7	4.1	1.3
	FQL-B	42.3	8.8	7.2	35.0	4.6	1.2

Unfermented Quinoa Gel (UQ-G), Fermented Quinoa Gel (FQ-G), Unfermented Lentil Gel (UL-G), Fermented Lentil Gel (FL-G), Unfermented Quinoa Lentil Gel (UQL-G), Fermented Quinoa Lentil Gel (FQL-G), Unfermented Lentil Bread (UL-B), Fermented Lentil Bread (FL-B), Unfermented Quinoa Lentil Bread (UQL-B), Fermented Quinoa Lentil Bread (FQL-B).

**Table 3:** Mean values of mechanical parameters for gels elaborated with fermented (F) and unfermented (U) flours of quinoa (Q) and lentil (L).

	TPA Test			Penetration Test		Cutting Test	
Prototypes	Hardness (N)	Elasticity	Cohesiveness	Force (N)	Distance (mm)	Force (N)	Adhesiveness
UQ-G	2.21 ^b,c^	0.90 ^a^	0.94 ^a,b^	6.84 ^a^	7.80 ^a^	2.40 ^a^	−0.67 ^a^
FQ-G	1.43 ^d^	0.90 ^a^	0.96 ^a^	4.18 ^d^	7.92 ^a^	1.25 ^d^	−0.56 ^a^
UL-G	2.05 ^c^	0.80 ^b^	0.89 ^c^	6.25 ^b^	4.63 ^c^	1.92 ^b,c^	−0.99 ^a,b^
FL-G	1.22 ^d^	0.81 ^b^	0.88 ^c^	3.36 ^e^	3.92 ^c^	1.38 ^d^	−1.30 ^b^
UQL-G	2.86 ^a^	0.89 ^a^	0.90 ^c^	5.98 ^b^	5.55 ^b^	2.15 ^b^	−1.01 ^a,b^
FQL-G	2.58 ^a,b^	0.88 ^a^	0.93 ^b^	5.49 ^c^	5.77 ^b^	1.79 ^c^	−1.24 ^b^

Unfermented Quinoa Gel (UQ-G), Fermented Quinoa Gel (FQ-G), Unfermented Lentil Gel (UL-G), Fermented Lentil Gel (FL-G), Unfermented Quinoa Lentil Gel (UQL-G), Fermented Quinoa Lentil Gel (FQL-G). For each parameter, values not sharing letters are significantly different according to Fisher test (*p* < 0.05).

**Table 4:** Mean values of mechanical parameters for breads elaborated with fermented (F) and unfermented (U) flours of quinoa (Q) and lentil (L).

	TPA Test			Penetration Test	
Prototypes	Hardness (N)	Elasticity	Cohesiveness	Force (N)	Distance (mm)
UQL-B	8.29 ^a,b^	0.81 ^a^	0.59 ^b^	0.89 ^b^	2.52 ^b^
FQL-B	5.88 ^b^	0.80 ^a^	0.71 ^a^	0.85 ^b^	3.56 ^a^
UL-B	6.34 ^b^	0.72 ^a^	0.50 ^c^	0.73 ^b^	1.76 ^c^
FL-B	9.96 ^a^	0.75 ^a^	0.69 ^a^	1.38 ^a^	3.57 ^a^

Unfermented Lentil Bread (UL-B), Fermented Lentil Bread (FL-B), Unfermented Quinoa Lentil Bread (UQL-B), Fermented Quinoa Lentil Bread (FQL-B). For each parameter, values not sharing letters are significantly different according to Fisher test (*p* < 0.05).

## Data Availability

Data will be made available upon reasonable request.
